# Mitochondrial genome of the critically endangered Baer’s Pochard, *Aythya baeri*, and its phylogenetic relationship with other Anatidae species

**DOI:** 10.1038/s41598-021-03868-7

**Published:** 2021-12-21

**Authors:** Dawei Liu, Yongwu Zhou, Yiling Fei, Chunping Xie, Senlin Hou

**Affiliations:** 1 Nanjing Forest Police College, Nanjing, 210023 China; 2 Key Laboratory for Forensic Technology of Wildlife, State Forestry and Grassland Administration, Nanjing, 210023 China; 3 College of Coastal Agricultural Sciences, Guangdong Ocean University, Zhanjiang, 524088 China

**Keywords:** Genetics, Molecular biology, Zoology

## Abstract

Historically, the diving duck, Baer’s Pochard (*Aythya baeri*) was widely distributed in East and South Asia, but according to a recent estimate, its global population is now less than 1000 individuals. To date, the mitochondrial genome of *A. baeri* has not been deposited and is not available in GenBank. Therefore, we aimed to sequence the complete mitochondrial genome of this species. The genome was 16,623 bp in length, double stranded, circular in shape, and contained 13 protein-coding genes, 22 tRNA genes, two rRNA genes, and one non-coding control region. Many structural and compositional similarities were discovered between *A. baeri* and the other three *Aythya* mitochondrial genomes. Among 13 protein-coding genes of the four *Aythya* species, the fastest-evolving gene was *ATP8* while the slowest-evolving gene was *COII.* Furthermore, the phylogenetic tree of Anatidae based on Bayesian inference and maximum likelihood methods showed that the relationships among 15 genera of the Anatidae family were as follows: *Dendrocygna* was an early diverging lineage that was fairly distant from the other ingroup taxa; *Cygnus*, *Branta*, and *Anser* were clustered into one branch that corresponded to the Anserinae subfamily; and *Aythya*, *Asarcornis*, *Netta*, *Anas*, *Mareca*, *Mergus*, *Lophodytes*, *Bucephala*, *Tadorna*, *Cairina*, and *Aix* were clustered into another branch that corresponded to the Anatinae subfamily. Our target species and three other *Aythya* species formed a monophyletic group. These results provide new mitogenomic information to support further phylogenetic and taxonomic studies and genetic conservation of Anatidae species.

## Introduction

The mitochondrion is a type of organelle. It has the ability to convert organic materials directly into energy to power the cell’s biological functions^1^. In multicellular animals, the mitochondrial genome (mitogenome) is a circular, double-stranded molecule with a closed structure. It usually has a length of ~ 16 kb and is made up of a heavy strand (H-strand) and a light strand (L-strand) containing a total of 37 genes: 13 protein-coding genes (PCGs), two ribosomal RNA genes (rRNAs), and 22 transfer RNA (tRNA) genes^2,3^. It also has one or two non-coding control regions (CR), known as A + T-rich regions^4^. Among the abovementioned genes in most birds, one PCG (*ND6*) and eight tRNA genes (*tRNA-Ala*, tRNA-Cys, *tRNA-Glu*, *tRNA-Gln*, *tRNA-Asn*, *tRNA-Pro*, *tRNA-Ser2*, and *tRNA-Tyr*) are located on the L-strand, whereas the CR and the remaining 28 genes are located on the H-strand^5,6^. Owing to its simple structure, maternal inheritance, small length, conserved gene sequences, and high evolutionary rate^7,8^ and its easy isolation, amplification, sequencing^9,10^, mitochondrial DNA (mtDNA) may be preferred to nuclear DNA for some applications that help elucidate population genetic diversity and molecular phylogenetic relationships^11,12^. Identifying and managing genetic diversity of a threatened species can remarkably help execute species recovery plans^13–15^. Thus, a basic step in analyzing variable molecular tags for conservation research is sequencing the complete mtDNA.

Baer’s Pochard (*Aythya baeri*) is a diving duck that belongs to the order Anseriformes, subfamily Anatinae, family Anatidae. Historically, it was distributed in the Amur and Ussuri River basins of far eastern Russia and northeastern China during the breeding season and in eastern and southern China, India, Bangladesh, and Myanmar following migration in winter^16^. It mainly feeds on insects and aquatic plants and animals^17^. In 2008, after a sharp decline in its population, it was classified as Endangered by the International Union for Conservation of Nature (IUCN), after which its conservation level increased to Critically Endangered in 2012^18^. The global population of Baer’s Pochard has been estimated to be less than 1000 individuals, but it could also be considerably lower^19^. Until the 1990s, major threats to its populations included habitat loss and degradation; recently, illegal hunting and disturbance may have become more significant factors causing its decline^20^. To protect this rare species from extinction, urgent and coordinated actions are needed.

The mitochondrial genome of *A. baeri* has not been deposited in GenBank so far, and molecular studies on this species are limited. In the current study, the complete mitogenome of this species was successfully sequenced. Based on the new sequence and previous data of other species, this study’s objectives were to address: (1) the characteristics of the mitogenome of *A. baeri*, (2) a comparison of the mitogenome of *A. baeri* and its relatives, (3) phylogenetic relationships of the family Anatidae on the basis of the combined mitochondrial gene set. These findings will help us better understand the *A. baeri* mitogenome and provide important information on the conservation and restoration of this endangered bird. Furthermore, the results could serve as a foundation for phylogenetic study as well as molecular biological data for taxonomic research on Anatidae species.

## Results and discussion

### Nucleotide composition and structure information

**Figure 1:**
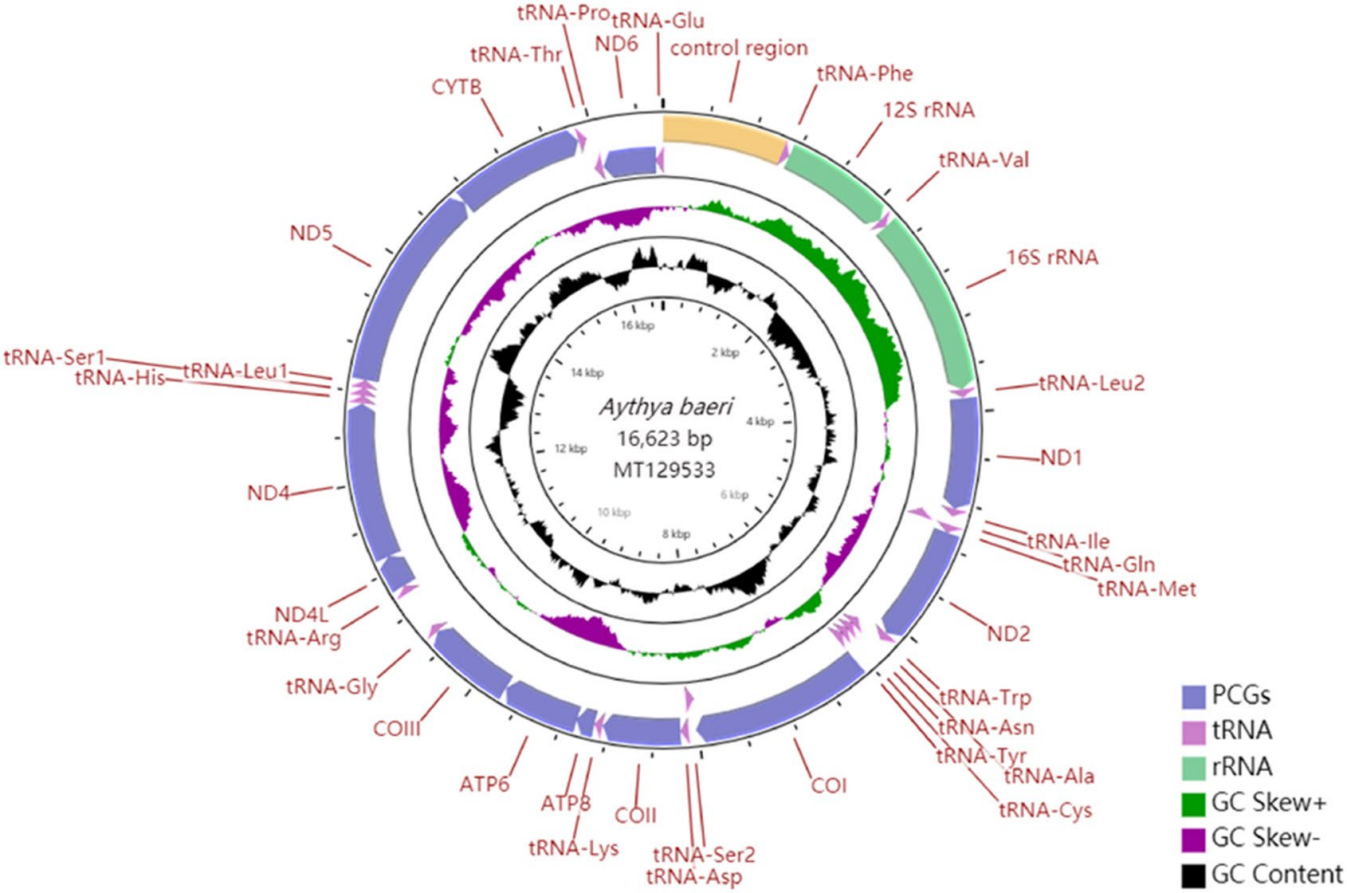
Circular map of the *Aythya baeri* mitochondrial genome. Different types of genes are indicated by squares of various colours. Genes encoded on the heavy or light strand are displayed on the outer or inner side of the circle, severally. The GC content of the mitogenome is represented by the black inner circles.

The entire mitochondrial genome of *A. baeri* was sequenced. It was deposited in GenBank under the accession number MT129533. Its size is 16,623 bp (Table 1). The lengths of the sequences discovered for other three *Aythya* mitogenomes were comparable at 16,616 bp. All the sizes were within the range of avian mitochondrial genomes^21^. As with the other animals^22,23^, mitogenome of *A. baeri* displayed a typical circular structure, including 13 PCGs, 22 tRNA genes, two rRNA genes, and one CR (Fig. 1). The arrangement and orientation of genes were similar to those found in other Anatidae species that had been determined^24^.

**Table 1:** Nucleotide compositions of the *Aythya* mitogenomes.

Species	Genome		PCGs		tRNAs		rRNAs		Control region	
	Size (bp)	AT (%)	Size (bp)	AT (%)	Size (bp)	AT (%)	Size (bp)	AT (%)	Size (bp)	AT (%)
*A. baeri*	16,623	51.95	11,403	50.94	1545	56.50	2587	53.54	1071	52.29
*A. americana*	16,616	51.62	11,406	50.60	1544	56.09	2586	53.21	1066	52.53
*A. ferina*	16,616	51.61	11,400	50.54	1545	56.31	2586	53.33	1055	52.51
*A. fuligula*	16,616	51.61	11,403	50.51	1544	56.54	2587	53.46	1054	51.90

**Figure 2:**
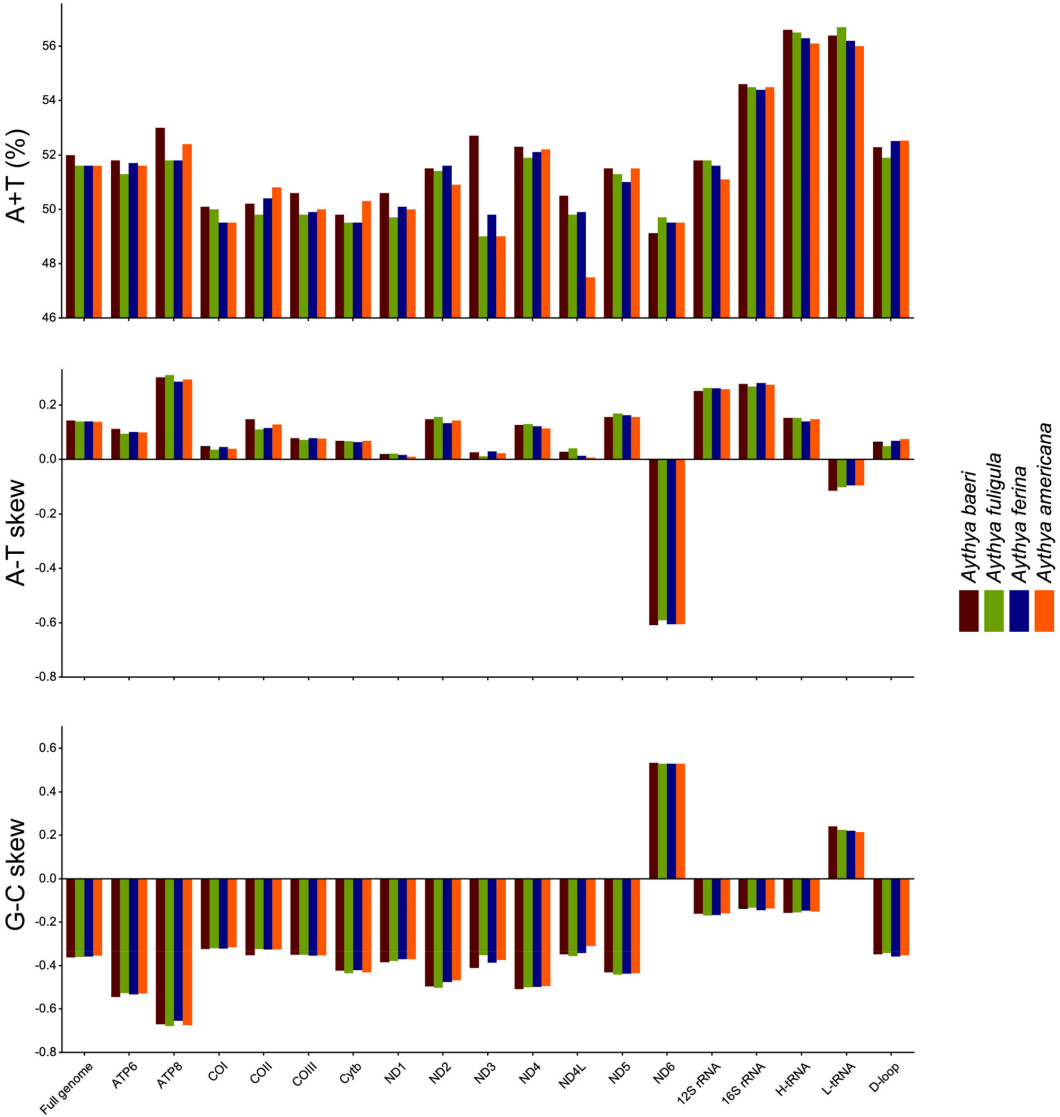
Nucleotide compositions of each gene and several genomic regions of four *Aythya* mitogenomes. The panels represent the AT- and GC-skews and the A + T content for every gene and several genomic areas within the mitogenome that are specified in the X-axis.

The parameters A + T content, AT-skew, and GC-skew are often employed to study the base composition pattern of mitogenomes^25^. The A + T content for *A. baeri* was 51.95%. It was slightly higher than that of other *Aythya* species genomes, which ranged from 51.61% to 51.62% (Table 1). In the full genome of *A. baeri*, the AT- and GC-skews were 0.143 and − 0.362, respectively. It demonstrated that the genome sequence was skewed away from T and G in favor of A and C, showing no difference from that of *A. americana*, *A. ferina*, and *A. fuligula* (Fig. 2). There were 35 bp of overlaps across the complete *A. baeri* mitogenome, with the greatest overlap having 10 bp between *ATP8* and *ATP6*. The *A. baeri* mitogenome had 12 intergenic spacers ranging in length from 1 to 10 bp, totaling 46 bp of non-coding nucleotides. The two longest spacers were located between *tRNA-Thr* and *tRNA-Pro* and *tRNA-Pro* and *ND6*, respectively (Table 2).

**Table 2:** Annotation of the whole mitogenome of *Aythya baeri*. Intergenic length is nucleotides after the named gene.

Gene	Strand	Location	Size (bp)	Intergenic length	Anticodon	Start codon	Stop codon
*CR*	H	1–1071	1071	0	–	–	–
*tRNA-Phe*	H	1072–1141	70	0	GAA	–	–
*12S rRNA*	H	1142–2124	983	0	–	–	–
*tRNA-Val*	H	2125–2195	71	0	TAC	–	–
*16S rRNA*	H	2196–3799	1604	0	–	–	–
*tRNA-Leu2*	H	3800–3873	74	4	TAA	–	–
*ND1*	H	3878–4855	978	− 2	–	ATG	AGG
*tRNA-Ile*	H	4854–4925	72	8	GAT	–	–
*tRNA-Gln*	L	4934–5004	71	− 1	TGG	–	–
*tRNA-Met*	H	5004–5072	69	0	CAT	–	–
*ND2*	H	5073–6113	1041	− 2	–	ATG	TAG
*tRNA-Trp*	H	6112–6187	76	3	TCA	–	–
*tRNA-Ala*	L	6191–6259	69	2	TGC	–	–
*tRNA-Asn*	L	6262–6334	73	0	GTT	–	–
*tRNA-Cys*	L	6335–6400	66	− 1	GCA	–	–
*tRNA-Tyr*	L	6400–6470	71	1	GTA	–	–
*COI*	H	6472–8022	1551	− 9	–	GTG	AGG
*tRNA-Ser2*	L	8014–8086	73	2	TGA	–	–
*tRNA-Asp*	H	8089–8157	69	1	GTC	–	–
*COII*	H	8159–8845	687	1	–	GTG	TAA
*tRNA-Lys*	H	8847–8914	68	1	TTT	–	–
*ATP8*	H	8916–9083	168	− 10	–	ATG	TAA
*ATP6*	H	9074–9757	684	− 1	–	ATG	TAA
*COIII*	H	9757–10,540	784	0	–	ATG	T-
*tRNA-Gly*	H	10,541–10,609	69	0	TCC	–	–
*ND3*	H	10,610–10,961	351	1	–	ATG	TAA
*tRNA-Arg*	H	10,963–11,033	71	0	TCG	–	–
*ND4L*	H	11,034–11,330	297	− 7	–	ATG	TAA
*ND4*	H	11,324–12,701	1378	0	–	ATG	T–
*tRNA-His*	H	12,702–12,770	69	0	GTG	–	–
*tRNA-Ser1*	H	12,771–12,836	66	− 1	GCT	–	–
*tRNA-Leu1*	H	12,836–12,906	74	0	TAG	–	–
*ND5*	H	12,907–14,730	1824	− 1	–	GTG	TAA
*Cytb*	H	14,730–15,872	1143	2	–	ATG	TAA
*tRNA-Thr*	H	15,875–15,943	69	10	TGT	–	–
*tRNA-Pro*	L	15,954–16,023	70	10	TGG	–	–
*ND6*	L	16,034–16,555	522	0	–	ATG	TAG
*tRNA-Glu*	L	16,556–16,623	68	0	TTC	–	–

### Protein-coding genes (PCGs) and codon usage

Among the four mitogenomes studied, only one gene, *ND6*, was located on the L-strand, whereas the remaining 12 genes were located on the H-strand (Fig. 1). Moreover, the entire size of the 13 PCGs of the four *Aythya* species ranged from 11,400 bp (*A. ferina*) to 11,406 bp (*A. americana*; Table 1). Furthermore, the average A + T content of the PCGs in each of the four *Aythya* species varied from 50.51% (*A. fuligula*) to 50.94% (*A. baeri*; Table 1). The AT- and GC-skew values of each PCG were similar among the four *Aythya* species (Fig. 2). Notably, all 12 genes located on the H-strand had biases with a positive AT-skew and a negative GC-skew, whereas the *ND6* gene located on the L-strand-encoded had the opposite biases.

Among the PCGs of *A. baeri*, the shortest PCG was *ATP8* that was found between *tRNA-Lys* and *ATP6*. In contrast, the longest PCG was *ND5* that was found between tRNA-Leu1 and *Cytb*. Except for *COI*, *COII*, and *ND5*, all of which had GTG as the start codon, all PCGs in the *A. baeri* mitogenome had a standard ATG start codon (Table 2). Seven genes (*COII*, *ATP6*, *ND3*, *ND4L*, *ND5*, *ATP8*, and *Cytb*) terminated with TAA. *ND1* and *COI* terminated with AGG, *ND2* and *ND6* terminated with TAG. Among the stop codons, TAA was the most common. *COIII* and *ND4* both had an incomplete T-stop codon.

**Figure 3:**
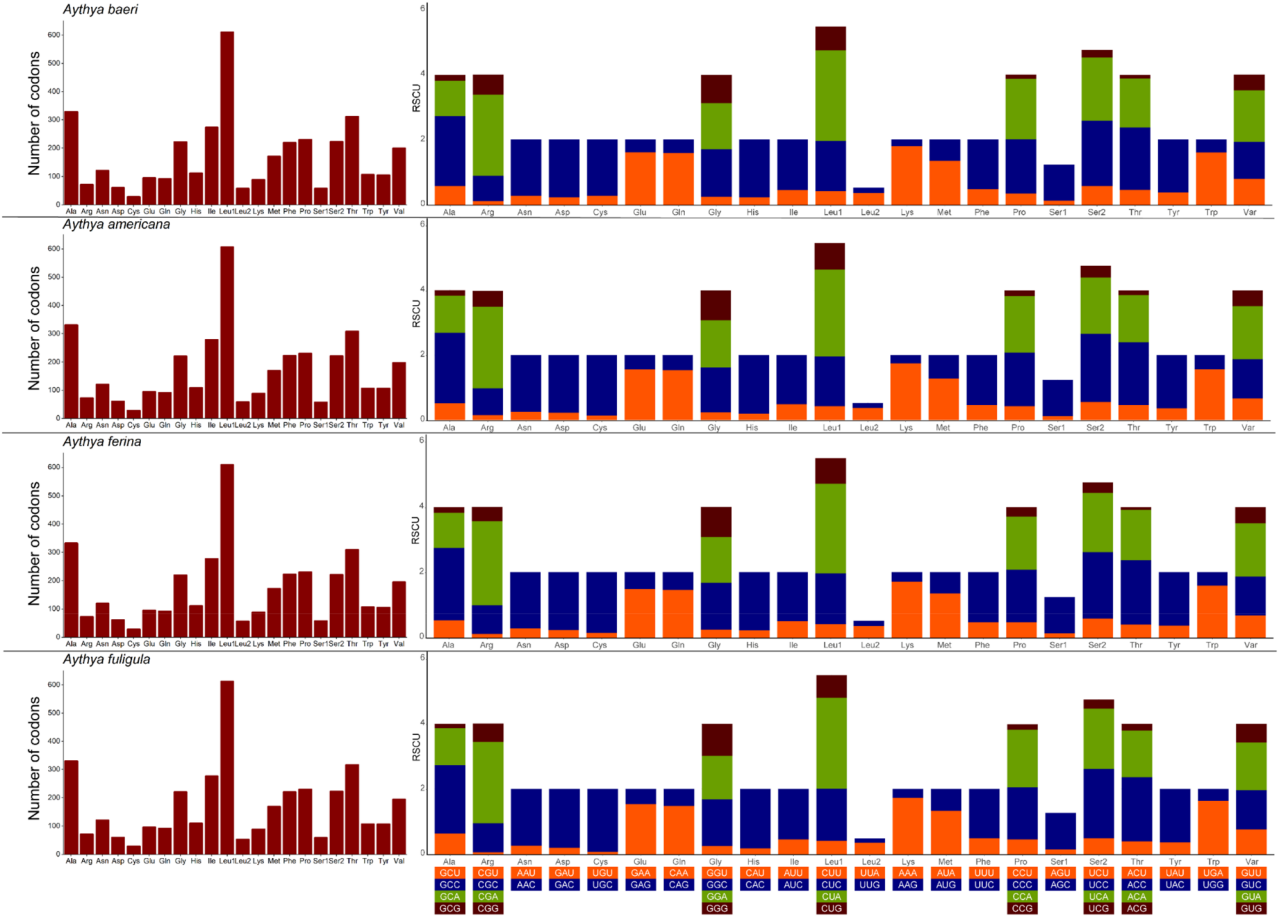
Codon distribution (left) and relative synonymous codon usage (right) in the mitochondrial protein-coding genes of four *Aythya* species.

Besides the stop codons, the PCGs contained 3800 to 3802 codons (Supplementary Tables 1–4) and showed similar codon distribution among the four *Aythya* species. In these mitogenomes, it can be found that codons encoding Leu1, Ala, Thr, Pro, and Ile were the most common. On the contrary, those encoding Cys were the least commonly observed (Fig. 3). In order to gain an insight into the genetic codon bias of the four *Aythya* species, the relative synonymous codon usage (RSCU) in PCG is shown in Fig. 3 and Supplementary Tables 1–4. The highly consistent RSCU was observed in the four birds, which could be due to their tight association as members of the same genus. It was also observed that for most amino acids, the use of synonymous codons was skewed. For example, in these species, the GCC codon for Ala was utilized frequently, whereas the GCG codon was rarely used for the same amino acid.

### Transfer and ribosomal RNA genes

**Figure 4:**
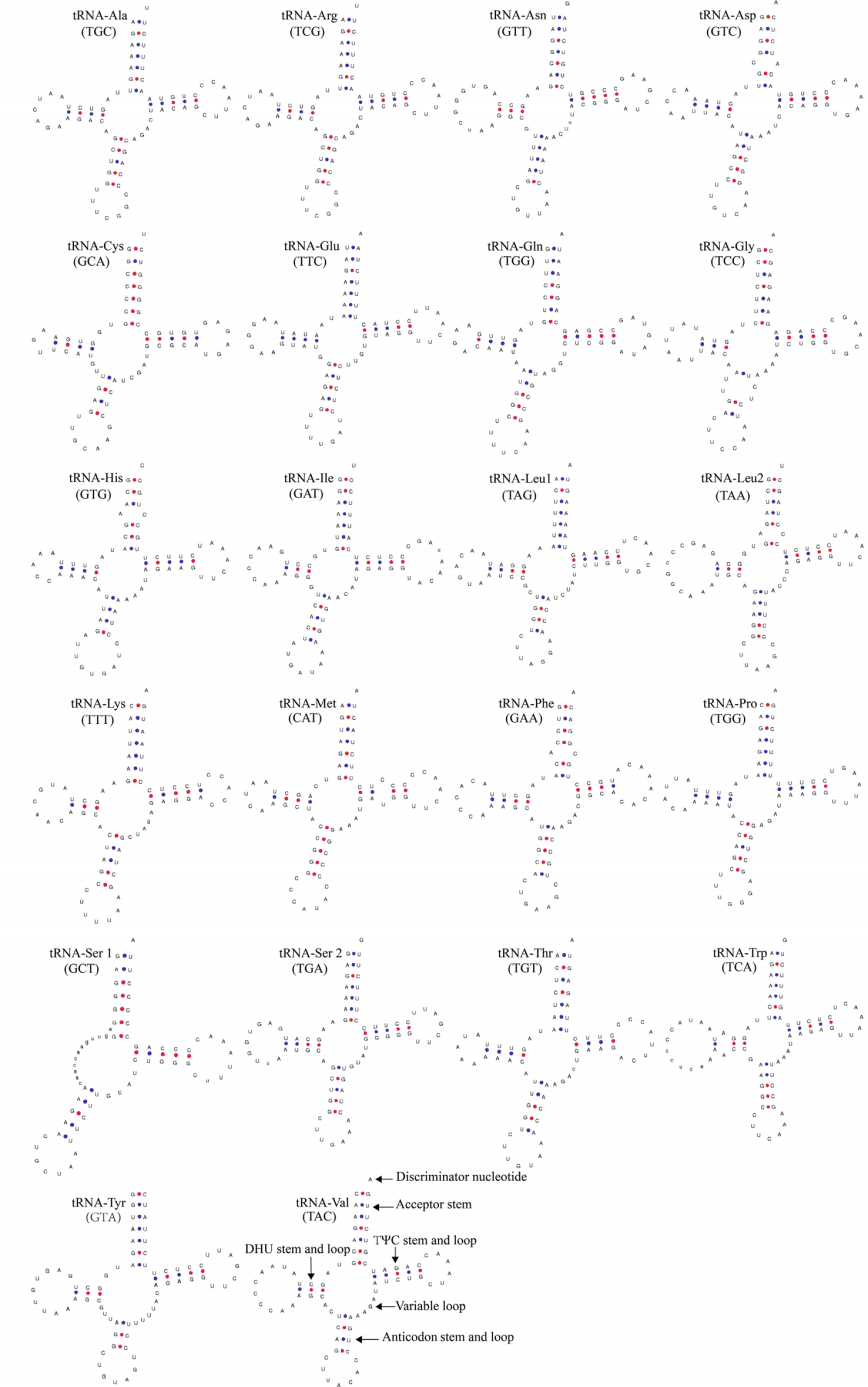
Secondary structures of *Aythya baeri* tRNA.

The *A. baeri* mtDNA contained 22 tRNA genes (Fig. 1 and Table 2) that ranged from 66 bp (*tRNA-Cys*) to 76 bp (*tRNA-Trp*). Among these tRNA genes, 14 genes were located on the H-strand and eight on the L-strand. As shown in Fig. 2, the tRNA genes located on the H-strand had positive and negative AT- and GC-skew values, respectively. However, the contrast was true for genes on the L-strand. Additionally, secondary structures of the 22 tRNAs were predicted (Fig. 4). Except for *tRNA-Ser1* lacking the dihydrouridine arm, almost all of the tRNA genes were found to possess a typical cloverleaf secondary structure. It is a characteristic generally observed in vertebrate tRNA genes^26^. In the mechanism of structural compensation between the other structures, it is possible that the missing arm in *tRNA-Ser1* is functional^27^.

Ribosomal RNA genes in the *A. baeri* mitochondrial genome include 12S rRNA and 16S rRNA. The two genes were found on the H-strand between *tRNA-Phe* and *tRNA-Leu2*, and separated by tRNA-Val, as in other animals^28,29^. The A + T content of these two genes among the four *Aythya* species varied from 56.09% to 56.54%. The AT- and GC-skew values for these two rRNA genes in each *Aythya* species were positive and negative, respectively (Fig. 2).

### Control region

The CR is thought to have transcription initiation sites as well as be involved in replication regulation in the mitochondrial genome^30^. In the field of phylogeny, this area has become a hot spot of research because of its rapid evolution and the largest variation in the whole mtDNA^31^. This CR was found to be located between *tRNA-Glu* and *tRNA-Phe*. The size of this region in *A. baeri* was 1071 bp (Table 1). This result was similar to the sequence lengths of the other three *Aythya* birds, ranging from 1054 bp (*A. fuligula*) to 1066 bp (*A. americana*)*.* The AT skews for the four species in CR varied from 0.0490 to 0.075 (Fig. 2). The GC skews varied from − 0.3412 to − 0.3573.

### Nucleotide diversity and selection pressures

**Figure 5:**
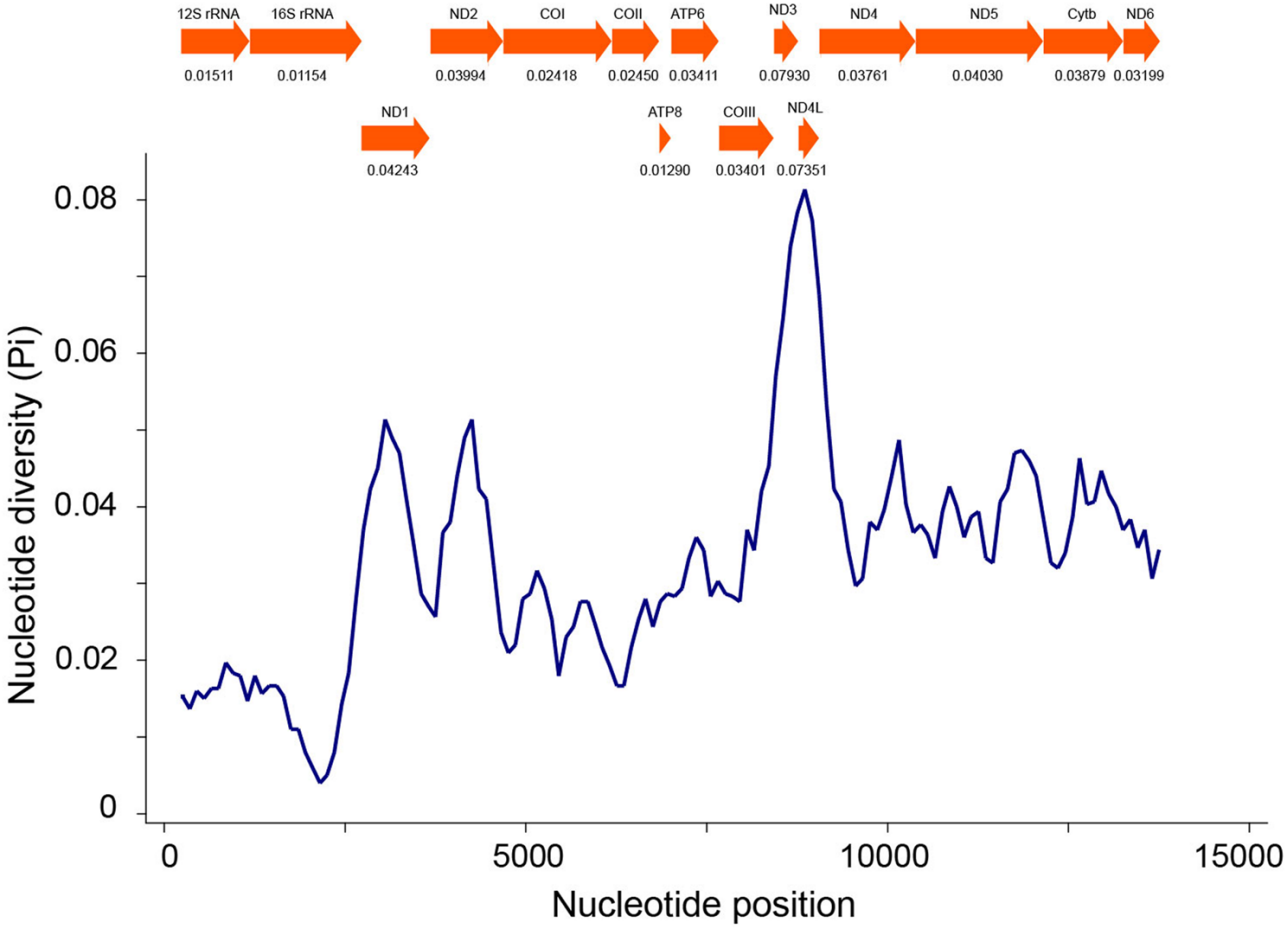
Sliding window analysis of PCGs and rRNA genes between four *Aythya* aves. The blue curve displays the value of nucleotide diversity (Pi). Each gene’s Pi value is displayed under the squares.

For the four ducks of *Aythya*, the sliding window analysis demonstrated that the nucleotide diversity levels differed dramatically among the PCGs and rRNA genes. As shown in Fig. 5, *ND3* (Pi = 0.0793) has the most polymorphism among the PCGs genes, followed by *ND4L* (Pi = 0.07351), *ND1* (Pi = 0.04243), and *ND5* (Pi = 0.0403). In contrast, *ATP8* (Pi = 0.0129), *COII* (Pi = 0.0254), and *COI* (Pi = 0.0418) had the lowest values and were the most conserved genes across the PCGs. Compared with most PCGs, *16S rRNA* (Pi = 0.01154) and *12S rRNA* (Pi = 0.01511) had relatively low variability.

**Figure 6:**
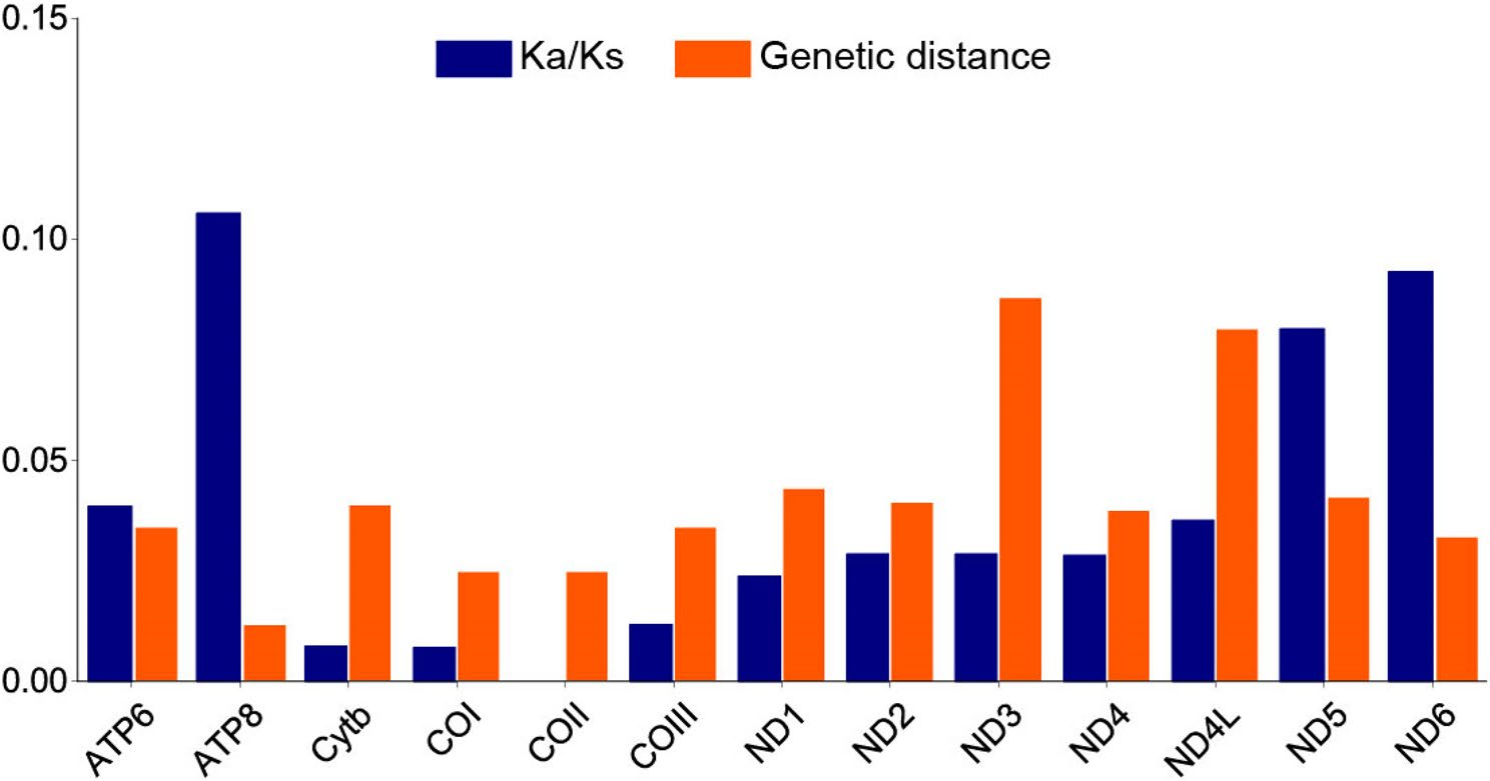
The ratios of Ka/Ks and genetic distances of each PCG among four *Aythya* aves.

The ratio of Ka/Ks is an effective parameter for indicating the selection pressure^32,33^. In order to analyze the pressure on mitochondrial PCGs, Ka/Ks ratios were evaluated for the four *Aythya* aves. As shown in Fig. 6, all the Ka/Ks ratios were lower than 1, demonstrating that the 13 PCGs are undergoing purifying selection. The Ka/Ks ratios were varied for each of the 13 PCGs, indicating differing amounts of functional constraints among the genes^34^. Specifically, the highest value was observed for *ATP8* (Ka/Ks = 0.10578), suggesting that *ATP8* faces the least amount of selection pressure and is the gene with the fastest rate of evolution. The lowest value was observed for *COII* (Ka/Ks = 0), reflecting that *COII* is subjected to the highest selective pressure and is the gene with the slowest evolution.

In order to estimate the average divergence among the four *Aythya* mtDNAs, the overall mean distances were analyzed based on 13 PCGs. As shown in Fig. 6, *ND3*, and *ND4L* appear to possess high distances of 0.087 and 0.080, followed by *ND1*, *ND5*, and *ND2*, while *ATP8*, *COII*, and *COI* have low distances of 0.013, 0.025, and 0.025, respectively. This tendency was similar to the nucleotide diversity levels of each PCG.

### Mitogenome phylogeny

The mitochondrial sequence is a widely used molecular marker for inferring phylogenetic estimations among animals^35^. In the present study, to further elucidate the phylogenetic interrelationships within Anatidae, concatenated nucleotide sequences of 13 PCGs and two rRNA genes were obtained from 37 Anatidae aves, concerning four *Aythya* species, one *Netta* species, one *Asarcornis* species, one *Mareca* species, eight *Anas* species, three *Mergus* species, one *Lophodytes* species, three *Bucephala* species, one *Cairina* species, one *Aix* species, two *Tadorna* species, four *Cygnus* species, one *Branta* species, five *Anser* species, and one *Dendrocygna* species.

**Figure 7:**
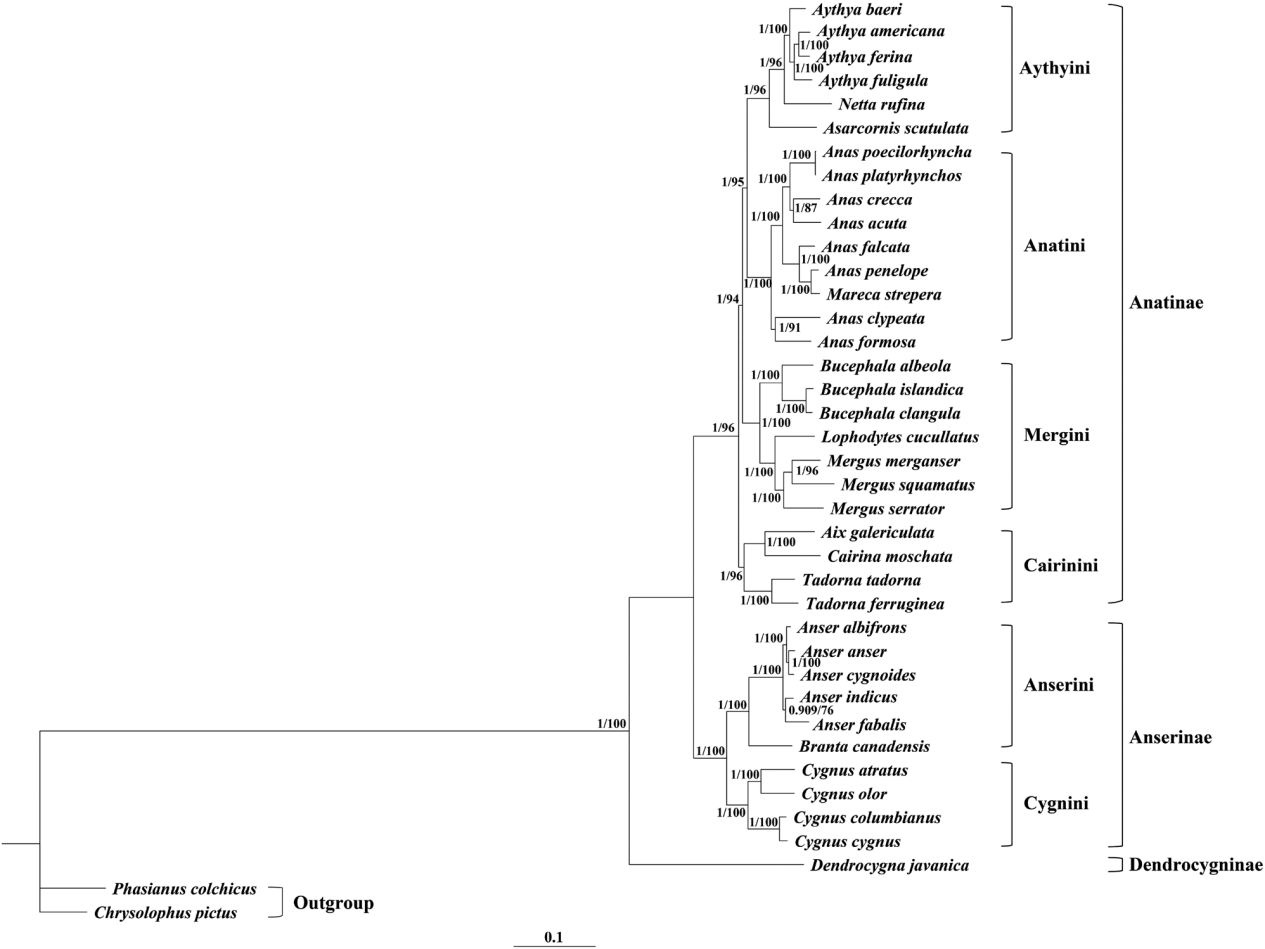
Molecular phylogenetic analysis of 37 Anatidae species constructed by MrBayes and IQ tree. Numbers beside the nodes represent posterior probabilities (left) and bootstrap values (right).

In both the Bayesian inference (BI) and maximum likelihood (ML) analyses, the phylogenetic trees had almost identical topologies (Fig. 7, Supplementary Figs. 1 and 2). Both trees from the two analyses were supported by good statistical values. In general, the genera *Aythya*, *Netta*, *Asarcornis*, *Anas*, *Mareca*, *Mergus*, *Lophodytes*, *Bucephala*, *Cairina*, *Tadorna*, and *Aix* clustered within the subfamily Anatinae, whereas the subfamily Anserinae had a sister relationship with Anatinae, including *Cygnus*, *Branta*, and *Anser*. *Dendrocygna javanica*, whose erect posture, elongated neck and legs, and conspicuous perching-tree habit distinguish it from most other Anseriformes species, diverged earlier from the main lineage and represents a sister clade to Anatinae-Anserinae^36^. Our topology was highly consistent with the results of studies based on the analysis of concatenated mitochondrial nucleotide sequences of *ND2* and *Cytb* genes, as well as with morphological data^36–38^.

Within Anserinae, species of the genus *Anser* are related to *Branta canadensis* closely. These taxa had a sister relationship with the *Cygnus* species assemblage. This is in accordance with previous morphology-based analyses^39^. The situation is more complex for the larger subfamily Anatinae. In this subfamily, we found four consistent clades: Aythyini (*Aythya* + *Netta* + *Asarcornis*), Anatini (*Anas* + *Mareca*), Mergini (*Mergus* + *Lophodytes* + *Bucephala*), and Cairinini (*Cairina* + *Aix* + *Tadorna*). Cairinini was the sister group of all other Anatinae tribes, which was strongly supported by the results of the present study. In the Anatinae tribes, Anatini and Aythyini have closer phylogenetic relationships, and these results are supported by morphological studies and phylogenetic analysis based on two PCGs^37,38^. Within the genus *Aythya*, *A. americana* and *A. ferina* were clustered together with well-supported values. Meanwhile, *A. fuligula* and the clade (*A. americana* + *A. ferina*) formed a high-supported value branch. Our target species, *A. baeri*, and the clade (*A. fuligula* + (*A. americana* + *A. ferina*)) were clustered into one branch, suggesting the four species form a monophyletic group.

## Conclusions

The current study provided the mitogenome of *A. baeri* (16,623 bp, accession number MT129533). This circular mtDNA included 13 PCGs, two rRNAs, 22 tRNAs, and one CR. Through comparison, it was found that the genome of *A. baeri* displayed characteristic sequence structures and was quite comparable to those of *Aythya* aves. Among the PCGs, *ND3* had the most polymorphism and highest genetic distance. Our phylogenetic analysis based on combined mitochondrial gene set (13 PCGs and two rRNAs) of 37 Anatidae species using BI and ML algorithms indicated that *A. baeri* is closely related to *A. fuligula*, *A. americana*, and *A. ferina*. In fact, we deduced that these species were monophyletic. Moreover, all the genera for which multiple species were studied were also discovered to be monophyletic with the exception of *Anas*, as that included *Mareca*. The present study provides an essential reference for the conservation of the studied critically endangered species as well as valuable information for further phylogenetic and evolutionary research on the family Anatidae.

## Materials and methods

### Sampling and DNA extraction

A sample of leg muscular tissue was obtained from a dead bird seized by the police in Sihong County, China, on October 27, 2019, no living animal was involved in this study. The sample was kept at minus 80 °C until DNA extraction. We extracted genomic DNA from the tissue using the Universal Genomic DNA Extraction Kit (Takara Biomedical Technology Co. Ltd., Beijing, China). The DNA quality was measured using agarose gel electrophoresis. DNA purity and concentration were assessed by NanoDrop 2000 (NanoDrop Technologies, Wilmington, NC, USA).

### Mitogenome sequencing and assembly

Following DNA extraction, we fragmented 1 μg of purified DNA and used it to set up 200 bp short-insert libraries. These qualified libraries were sequenced with PE150 bp on an BGISEQ-500 sequencer according to the manufacturer’s instructions detailed in the previous literature^40^. As a result, we obtained 5.2 Gb of clean data and trimmed all the raw reads using FASTQ^41^. Subsequently, we mapped the high-quality reads using default parameters to reference *Aythya* mitogenome datasets (accession numbers: AF090337, KJ710708, KJ722069), which we obtained from GenBank through Bowtie^42^. The sequence of the coding gene having the maximum coverage was then utilized as a seed sequence for de novo assembly of the mitochondrial genome NOVOPlasty^43^. Once the assembly was completed, we re-mapped all the sequencing reads to the candidate mitochondrial genome to re-confirm the assembly of the mitochondrial genome.

### Mitogenome annotation and analysis

All PCGs were first identified utilizing the NCBI website’s open reading frame finder^44^. Then, MEGA 7.0 was used to translate the sequences into putative proteins^45^. Verifications of tRNA genes were performed using the MITOS WebServer^46^, and secondary structures were inferred using tRNAscan-SE^47^. Sequences of the identified genes were re-checked by comparing them with that of other *Aythya* species manually. A map of the *A. baeri* mitochondrial genome was generated by CGView^48^. MEGA 7.0 was used to analyze the base compositions and RSCU values of 13 PCGs^45^. To estimate the bias in nucleotide composition across the entire sequence, AT- and GC-skews were used. This was obtained based on the formulas: AT skew = (A − T)/(A + T) and GC skew = (G − C)/(G + C)^49^. For the four *Aythya* aves, the nucleotide diversity (Pi) of each PCG and rRNA, a sliding window analysis in 500 bp windows every 100 bp of 13 PCGs and two rRNAs, the ratios of non-synonymous (Ka) to synonymous (Ks) substitutions rates of each PCG were conducted by DnaSP 5.0^50^. MEGA 7.0 was used to compute the overall mean distances among the four *Aythya* species, using the Kimura-2-parameter model^45^.

### Phylogenetic analysis

The phylogenetic status of *A. baeri* was assessed by comparing the combined mitochondrial gene set (13 PCGs and two rRNA genes) with that of 36 other Anatidae species across three subfamilies: Anatinae, Anserinae, and Dendrocygninae, and 15 genera: *Aythya*, *Asarcornis*, *Netta*, *Anas*, *Mareca*, *Mergus*, *Lophodytes*, *Bucephala*, *Tadorna*, *Cairina*, *Aix*, *Cygnus*, *Branta*, *Anser*, and *Dendrocygna* (Table 3). In addition, *Phasianus colchicus* and *Chrysolophus pictus* were set as outgroups for rooting the tree. GenBank was used to obtain all the mitogenome sequences.

**Table 3:** Taxonomic information used in the phylogenetic analysis.

Subfamily	Genus	Species	Genbank No.
Anatinae	*Aythya*	*Aythya americana*	AF090337
		*Aythya baeri*	MT129533
		*Aythya ferina*	KJ710708
		*Aythya fuligula*	KJ722069
	*Netta*	*Netta rufina*	KC466568
	*Asarcornis*	*Asarcornis scutulata*	MN356440
	*Anas*	*Anas falcata*	NC_023352
		*Anas poecilorhyncha*	KC466567
		*Anas platyrhynchos*	EU009397
		*Anas crecca*	KF203133
		*Anas acuta*	KF312717
		*Anas clypeata*	NC_028346
		*Anas penelope*	MT304825
		*Anas formosa*	JF730435
	*Mareca*	*Mareca strepera*	MN186586
	*Mergus*	*Mergus squamatus*	HQ833701
		*Mergus merganser*	KU140667
		*Mergus serrator*	MW849285
	*Lophodytes*	*Lophodytes cucullatus*	MW849287
	*Bucephala*	*Bucephala albeola*	MW849286
		*Bucephala islandica*	MW849281
		*Bucephala clangula*	MW849283
	*Cairina*	*Cairina moschata*	EU755254
	*Aix*	*Aix galericulata*	KJ169568
	*Tadorna*	*Tadorna ferruginea*	KF684946
		*Tadorna tadorna*	KU140668
Anserinae	*Cygnus*	*Cygnus olor*	KP981364
		*Cygnus atratus*	FJ379295
		*Cygnus cygnus*	KP981363
		*Cygnus columbianus*	DQ083161
	*Branta*	*Branta canadensis*	NC_007011
	*Anser*	*Anser cygnoides*	KY767671
		*Anser albifrons*	MH000287
		*Anser indicus*	KM455570
		*Anser fabalis*	HQ890328
		*Anser anser*	MN122908
Dendrocygninae	*Dendrocygna*	*Dendrocygna javanica*	FJ379296
Phasianinae	*Phasianus*	*Phasianus colchicus*	NC_015526
	*Chrysolophus*	*Chrysolophus pictus*	FJ752433

The sequences of the gene set of mitogenomes of 37 species were combined, and MAFFT was used to generate the alignment of the concatenated genes^51^. Gblocks was used to select conserved sequences within the database. Using the method described by Xia et al.^52^, DAMBE was used to perform a substitution saturation analysis for dataset.

The results showed that the dataset was suitable for further analyses. BI and ML methods were applied to phylogenetic analysis. Using ModelFinder^53^, it was found that the GTR + F + I + G4 model was the best-fit model for the BI and ML methods based on the AIC^54^ and BIC^55^, respectively. The BI was performed by MrBayes^56^ with two simultaneous MCMC chains, running for twenty million cycles, sampling every 2000 generations, and discarding the initial 0.25 of sampled data as burn-in. Trace plot in Tracer v1.7 was used to assess convergence. The effective sample size of model parameters was much more than 200. The ML analysis was run with standard bootstrap for 1000 replicates using IQ-TREE^57^. Both resulting phylograms were visualized and edited in FigTree 1.4.4^58^.

## Supplementary Information

Supplementary Information.

## Data Availability

The mitochondrial genome data has been submitted to NCBI GenBank under the following Accession Numbers MT129533.
