# Supplementary material for: Mitochondrial genome of the critically endangered Baer’s Pochard, Aythya baeri, and its phylogenetic relationship with other Anatidae species
Source: Sci Rep. 2021 Dec 21;11:24302. doi: 10.1038/s41598-021-03868-7 (PMC8692624; doi:10.1038/s41598-021-03868-7)
Supplement: Supplementary file 1 — Supplementary Information. [file 41598_2021_3868_MOESM1_ESM.pdf]

Supplementary data

Mitochondrial genome of the critically endangered Baer's Pochard, *Aythya baeri*, and its phylogenetic relationship with other Anatidae species

Dawei Liu [1,2], Yongwu Zhou [1,2], Yiling Fei [1,2], Chunping Xie [3] and Senlin Hou [1,2*]

[1]Nanjing Forest Police College, Nanjing 210023, China; dwliu@nfpc.edu.cn (D.L.); ywzhou@nfpc.edu.cn (Y.Z.); ylfei@nfpc.edu.cn (Y.F.)

[2]Key Laboratory for Forensic Technology of Wildlife, State Forestry and grassland administration, Nanjing 210023, China

[3]College of Coastal Agricultural Sciences, Guangdong Ocean University, Zhanjiang 524088, China; xcp@gdou.edu.cn

*Correspondence author. E-mail address: slhou@nfpc.edu.cn; Tel.: +86-025-85878854

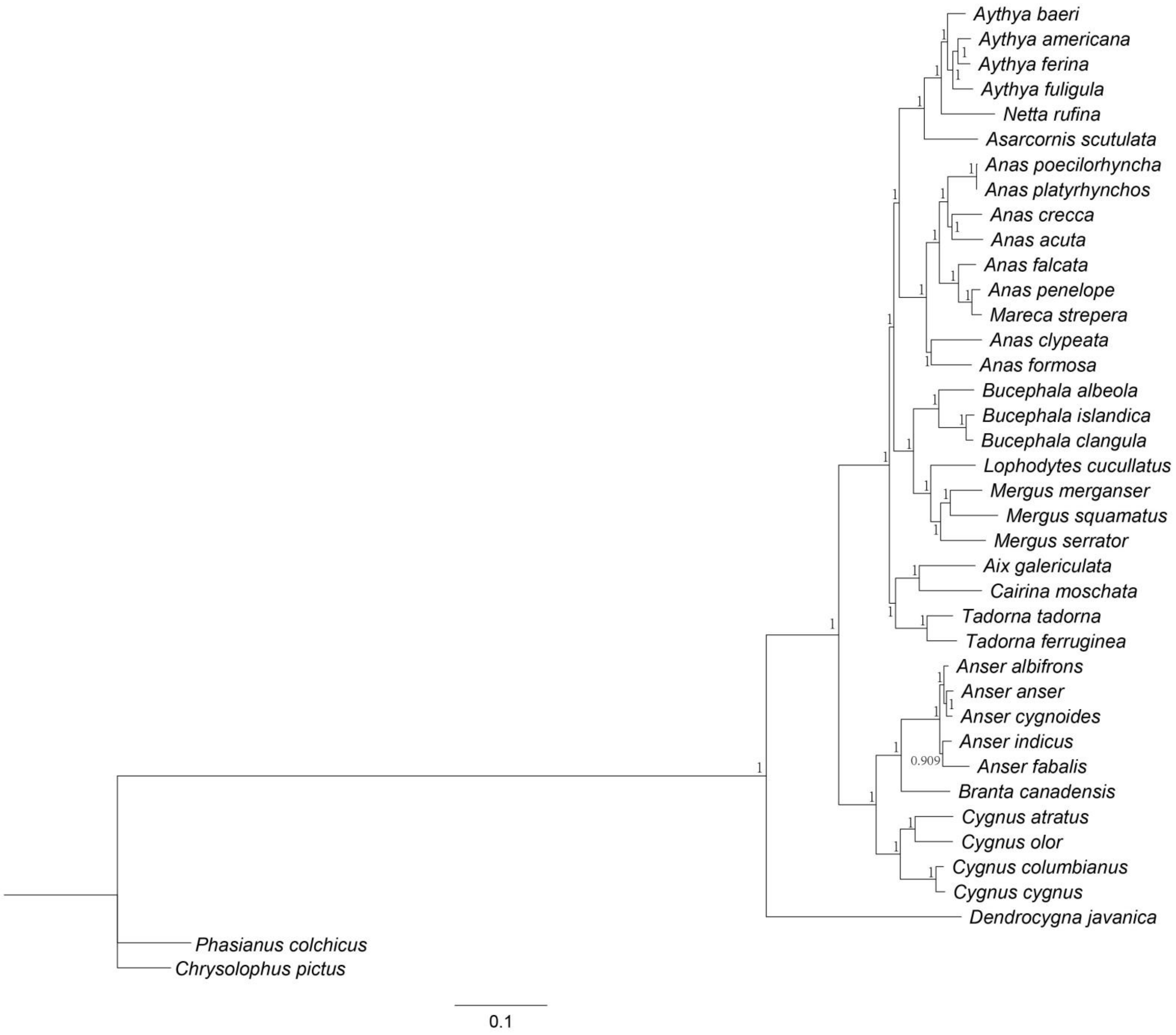

Supplementary Figure 1. Phylogenetic tree inferred from nucleotide sequences of 13 PCGs and two rRNAs using the BI analysis.

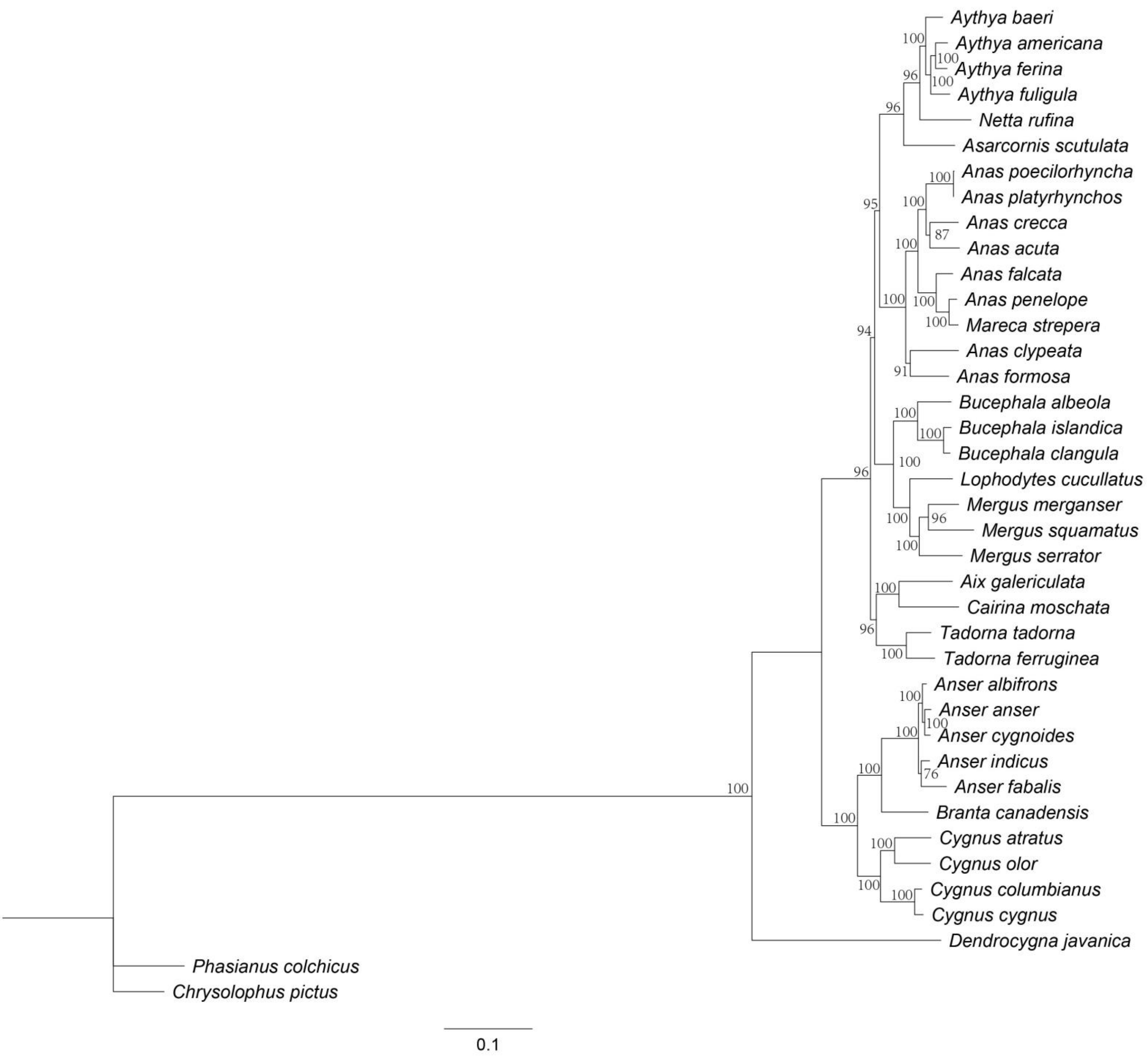

Supplementary Figure 2. Phylogenetic tree inferred from nucleotide sequences of 13 PCGs and two rRNAs using the ML analysis.

Supplementary Table 1. Codon number and RSCU of *Aythya baeri* mitochondrial PCGs.

**Codon**	**Count**	**RSCU**	**Codon**	**Count**	**RSCU**	**Codon**	**Count**	**RSCU**	**Codon**	**Count**	**RSCU**
UUU(F)	53	0.48	UCU(S)	27	0.58	UAU(Y)	20	0.38	UGU(C)	4	0.28
UUC(F)	167	1.52	UCC(S)	94	2.01	UAC(Y)	85	1.62	UGC(C)	25	1.72
UUA(L)	41	0.37	UCA(S)	91	1.94	UAA(*)	7	2.55	UGA(W)	86	1.61
UUG(L)	17	0.15	UCG(S)	11	0.23	UAG(*)	2	0.73	UGG(W)	21	0.39
CUU(L)	47	0.42	CCU(P)	20	0.35	CAU(H)	13	0.23	CGU(R)	2	0.11
CUC(L)	172	1.54	CCC(P)	96	1.68	CAC(H)	99	1.77	CGC(R)	14	0.78
CUA(L)	311	2.79	CCA(P)	106	1.85	CAA(Q)	73	1.59	CGA(R)	45	2.50
CUG(L)	81	0.73	CCG(P)	7	0.12	CAG(Q)	19	0.41	CGG(R)	11	0.61
AUU(I)	62	0.45	ACU(T)	36	0.46	AAU(N)	17	0.28	AGU(S)	6	0.13
AUC(I)	212	1.55	ACC(T)	150	1.92	AAC(N)	104	1.72	AGC(S)	52	1.11
AUA(M)	115	1.35	ACA(T)	118	1.51	AAA(K)	80	1.80	AGA(*)	0	0
AUG(M)	56	0.65	ACG(T)	8	0.1	AAG(K)	9	0.20	AGG(*)	2	0.73
GUU(V)	40	0.8	GCU(A)	48	0.58	GAU(D)	7	0.23	GGU(G)	14	0.25
GUC(V)	57	1.14	GCC(A)	177	2.15	GAC(D)	54	1.77	GGC(G)	81	1.46
GUA(V)	79	1.58	GCA(A)	90	1.09	GAA(E)	77	1.62	GGA(G)	79	1.42
GUG(V)	24	0.48	GCG(A)	14	0.17	GAG(E)	18	0.38	GGG(G)	48	0.86

Supplementary Table 2. Codon number and RSCU of *Aythya americana* mitochondrial PCGs.

Codon	Count	RSCU	Codon	Count	RSCU	Codon	Count	RSCU	Codon	Count	RSCU
UUU(F)	52	0.47	UCU(S)	26	0.56	UAU(Y)	20	0.37	UGU(C)	2	0.14
UUC(F)	171	1.53	UCC(S)	98	2.1	UAC(Y)	87	1.63	UGC(C)	27	1.86
UUA(L)	42	0.38	UCA(S)	81	1.74	UAA(*)	7	2.55	UGA(W)	84	1.57
UUG(L)	17	0.15	UCG(S)	17	0.36	UAG(*)	2	0.73	UGG(W)	23	0.43
CUU(L)	48	0.43	CCU(P)	25	0.43	CAU(H)	11	0.2	CGU(R)	3	0.16
CUC(L)	170	1.53	CCC(P)	95	1.65	CAC(H)	98	1.8	CGC(R)	15	0.82
CUA(L)	298	2.68	CCA(P)	101	1.75	CAA(Q)	71	1.54	CGA(R)	46	2.52
CUG(L)	91	0.82	CCG(P)	10	0.17	CAG(Q)	21	0.46	CGG(R)	9	0.49
AUU(I)	69	0.49	ACU(T)	36	0.47	AAU(N)	16	0.26	AGU(S)	6	0.13
AUC(I)	210	1.51	ACC(T)	149	1.93	AAC(N)	105	1.74	AGC(S)	52	1.11
AUA(M)	109	1.28	ACA(T)	113	1.46	AAA(K)	78	1.75	AGA(*)	0	0
AUG(M)	61	0.72	ACG(T)	11	0.14	AAG(K)	11	0.25	AGG(*)	2	0.73
GUU(V)	33	0.67	GCU(A)	43	0.52	GAU(D)	7	0.23	GGU(G)	13	0.24
GUC(V)	60	1.21	GCC(A)	180	2.18	GAC(D)	54	1.77	GGC(G)	77	1.39
GUA(V)	81	1.64	GCA(A)	94	1.14	GAA(E)	74	1.56	GGA(G)	80	1.45
GUG(V)	24	0.48	GCG(A)	14	0.17	GAG(E)	21	0.44	GGG(G)	51	0.92

Supplementary Table 3. Codon number and RSCU of *Aythya ferina* mitochondrial PCGs.

Codon	Count	RSCU	Codon	Count	RSCU	Codon	Count	RSCU	Codon	Count	RSCU
UUU(F)	52	0.47	UCU(S)	27	0.58	UAU(Y)	19	0.36	UGU(C)	2	0.14
UUC(F)	171	1.53	UCC(S)	95	2.04	UAC(Y)	87	1.64	UGC(C)	27	1.86
UUA(L)	39	0.35	UCA(S)	85	1.82	UAA(*)	7	3.11	UGA(W)	85	1.59
UUG(L)	18	0.16	UCG(S)	15	0.32	UAG(*)	0	0	UGG(W)	22	0.41
CUU(L)	45	0.41	CCU(P)	27	0.47	CAU(H)	12	0.22	CGU(R)	2	0.11
CUC(L)	173	1.56	CCC(P)	93	1.61	CAC(H)	99	1.78	CGC(R)	16	0.88
CUA(L)	305	2.75	CCA(P)	94	1.63	CAA(Q)	67	1.46	CGA(R)	47	2.58
CUG(L)	86	0.77	CCG(P)	17	0.29	CAG(Q)	25	0.54	CGG(R)	8	0.44
AUU(I)	70	0.5	ACU(T)	31	0.4	AAU(N)	17	0.28	AGU(S)	6	0.13
AUC(I)	208	1.5	ACC(T)	153	1.97	AAC(N)	103	1.72	AGC(S)	52	1.11
AUA(M)	116	1.35	ACA(T)	120	1.55	AAA(K)	76	1.71	AGA(*)	0	0
AUG(M)	56	0.65	ACG(T)	6	0.08	AAG(K)	13	0.29	AGG(*)	2	0.89
GUU(V)	33	0.68	GCU(A)	44	0.53	GAU(D)	7	0.23	GGU(G)	13	0.24
GUC(V)	58	1.19	GCC(A)	185	2.22	GAC(D)	54	1.77	GGC(G)	79	1.44
GUA(V)	80	1.64	GCA(A)	90	1.08	GAA(E)	71	1.49	GGA(G)	77	1.4
GUG(V)	24	0.49	GCG(A)	14	0.17	GAG(E)	24	0.51	GGG(G)	51	0.93

Supplementary Table 4. Codon number and RSCU of *Aythya fuligula* mitochondrial PCGs.

**Codon**	**Count**	**RSCU**	**Codon**	**Count**	**RSCU**	**Codon**	**Count**	**RSCU**	**Codon**	**Count**	**RSCU**
UUU(F)	54	0.49	UCU(S)	23	0.49	UAU(Y)	19	0.36	UGU(C)	1	0.07
UUC(F)	167	1.51	UCC(S)	100	2.13	UAC(Y)	88	1.64	UGC(C)	28	1.93
UUA(L)	39	0.35	UCA(S)	87	1.85	UAA(*)	7	2.8	UGA(W)	88	1.64
UUG(L)	14	0.13	UCG(S)	13	0.28	UAG(*)	1	0.4	UGG(W)	19	0.36
CUU(L)	46	0.41	CCU(P)	26	0.45	CAU(H)	10	0.18	CGU(R)	1	0.06
CUC(L)	178	1.6	CCC(P)	92	1.6	CAC(H)	100	1.82	CGC(R)	16	0.89
CUA(L)	310	2.79	CCA(P)	102	1.77	CAA(Q)	68	1.48	CGA(R)	45	2.5
CUG(L)	79	0.71	CCG(P)	10	0.17	CAG(Q)	24	0.52	CGG(R)	10	0.56
AUU(I)	63	0.45	ACU(T)	31	0.39	AAU(N)	16	0.26	AGU(S)	7	0.15
AUC(I)	214	1.55	ACC(T)	157	1.98	AAC(N)	105	1.74	AGC(S)	52	1.11
AUA(M)	112	1.33	ACA(T)	113	1.43	AAA(K)	77	1.73	AGA(*)	0	0
AUG(M)	57	0.67	ACG(T)	16	0.2	AAG(K)	12	0.27	AGG(*)	2	0.8
GUU(V)	37	0.76	GCU(A)	52	0.63	GAU(D)	6	0.2	GGU(G)	14	0.25
GUC(V)	59	1.21	GCC(A)	173	2.1	GAC(D)	54	1.8	GGC(G)	79	1.43
GUA(V)	71	1.46	GCA(A)	94	1.14	GAA(E)	74	1.54	GGA(G)	74	1.34
GUG(V)	28	0.57	GCG(A)	11	0.13	GAG(E)	22	0.46	GGG(G)	54	0.98
